# Skin under Strain: From Epithelial Model Tissues to Adult Epithelia

**DOI:** 10.3390/cells10071834

**Published:** 2021-07-20

**Authors:** Robin Püllen, Jens Konrad, Rudolf Merkel, Bernd Hoffmann

**Affiliations:** Forschungszentrum Jülich, Institute of Biological Information Processing, IBI-2: Mechanobiology, 52428 Jülich, Germany; r.puellen@fz-juelich.de (R.P.); j.konrad@fz-juelich.de (J.K.); r.merkel@fz-juelich.de (R.M.)

**Keywords:** epithelia, skin, epidermis equivalent, biomimetic systems, tissue stretching, strain, mechanoadaptation, tensile test, viscoelasticity, elastin

## Abstract

Formation of a barrier capable of protecting tissue from external damage, chemical factors, and pathogens is one of the main functions of the epidermis. Furthermore, upon development and during aging, mechanoprotective epidermal functions change dramatically. However, comparative studies between embryonic and adult skin in comparison to skin equivalents are still scarce which is especially due to the lack of appropriate measurement systems with sufficient accuracy and long-term tissue compatibility. Our studies fill this gap by developing a combined bioreactor and tensile testing machine for biomechanical analysis of living epithelia. Based on this tissue stretcher, our data clearly show that viscoelastic and plastic deformation behavior of embryonic and adult skin differ significantly. Tissue responses to static strain compared to cyclic strain also show a clear dependence on differentiation stage. Multilayered unkeratinized epidermis equivalents, on the other hand, respond very similar to mechanical stretch as adult tissue. This mechanical similarity is even more evident after a single cycle of mechanical preconditioning. Our studies therefore suggest that skin equivalents are well suited model systems to analyze cellular interactions of epidermal cells in natural tissues.

## 1. Introduction

Epithelia separate organs and tissues from one another and from the environment. Hereby they act as physical barriers with protective and regulatory functions. The skin, in particular, is permanently affected by mechanical stress and had to develop structures and mechanisms to preserve integrity [1]. Mature skin consists of two major layers: the epidermis and dermis. Occasionally, hypodermal tissue is added as a third skin layer. The epidermis is located superficially. It is assembled as stratified epithelium that is composed of tightly connected cells, namely keratinocytes, which are linked to the underlying dermal tissue via a basement membrane [2]. The dermis, on the other hand, is a dense connective tissue consisting of fibrillary proteins embedded in a gel-like ground substance and approximately 20 times thicker than the epidermis [3]. Finally, the hypodermis underneath is mainly made of fat-storing cells and serves as isolation and loose connection to cartilage and bones [4].

From an engineering point of view, mature skin is a highly non-linear, anisotropic and viscoelastic material [5,6]. While anisotropy leads to the well-described Langer’s lines as well as to an impact of sample orientation and location on elastic properties, viscoelasticity causes strain rate dependency of mechanical properties and the typical stress relaxation under prolonged loading [7,8]. The highly non-linear stress-strain curves measured in tensile testing are often described as “J shaped” and originate from dermal microstructure [9,10]. In the initial loading phase, the so-called ”toe region”, the material response, is very soft, and relatively high strain levels are reached with only small increase of tissue stress. Subsequently, fibrillary proteins are gradually oriented (heel region) and tightened (linear region). During this sequence, material stiffness increases by orders of magnitude and the material displays tremendous levels of strain stiffening [6,11]. The mechanical properties of mature skin are most often discussed in terms of microstructure and properties of the collagen network in the dermis [12]. This was convincingly demonstrated by electron microscopy and X-ray diffraction on skin at intermediate and high strain levels where collagen fibers progressively straightened and aligned to the direction of strain to be finally separated into fibrils shortly before failure [13]. At lower strain levels, collagen fibers stay largely wavy and randomly orientated [11]. Here, elastin and a gel-like matrix primarily made of glycosaminoglycans are considered to be responsible for the mechanical properties [14]. All layers of the skin, but especially the dermis, develop extensively in a late fetal stage but also postnatally to their mature structure and composition. Cornification of the epidermis, decellularization of dermal tissue, cross-linking of collagen, and emergence of elastin should be mentioned here exemplarily [15]. In general, knowledge on the mechanical properties of developing skin is scarce and, to the best of our knowledge, no mechanical study directly compared fetal and mature tissue.

Additionally, despite intense research, the complex mechanical response of mature full-thickness skin under physiological conditions is poorly understood. This holds especially true at comparatively small strains. In general, in vivo measurements are performed in this small strain regime but they are often difficult to reproduce whereas measurement devices for in vitro mechanical testing almost invariably perform much better in the high strain and high force regime [16,17,18]. Therefore, many publications focused on excised human or animal tissue at high and possibly non-physiological stress levels to characterize the mechanical properties of skin [10,19,20,21]. Here, monotonic or cyclic uniaxial tensile tests were used to analyze collagen fiber orientation and fracture as well as the influence of strain rate on viscoelasticity and anisotropy. Unfortunately, many publications on ex vivo skin samples give little or no information on sample conditions even though hydration has been shown to strongly influence mechanical behavior [11,14,22]. In addition, cell viability was rarely explored. The role of cells for the mechanics of skin remains therefore largely obscure. This becomes even more important since homeostasis of the skin, as well as wound repair and pathogenesis of diseases, are particularly governed by intrinsic and extrinsic forces [23].

As an alternative for ex vivo skin samples, many biomedical studies use isolated cells or epithelial cell monolayers to investigate mutations accounting for a variety of diseases like epidermolysis bullosa [24,25]. To improve on the highly simplified and therefore artificial character of monolayers, multilayered skin equivalents can be formed [26,27]. Such systems seem to largely mimic natural epidermal conditions. However, due to their dependence on rather stiff adhesion substrates and cultivation at the air-liquid interface, detailed mechanical characterization of such systems is still lacking [28]. With ensured mechanical similarities, these model systems would greatly enhance our understanding of epidermal mechanics and cellular processes within.

For this reason, we developed a highly sensitive, environmental-controlled tensile testing machine. This instrument, which we call “tissue stretcher” for simplicity reasons, allowed, in a first direct approach, to evaluate and optimally compare the mechanical properties of a cell culture-based, multilayered epidermis model system with native tissue. Besides full-thickness adult rat skin, we used skin samples of 18-day-old rat embryos.

## 2. Materials and Methods

### 2.1. Tissue Stretcher Design

**Figure 1:**
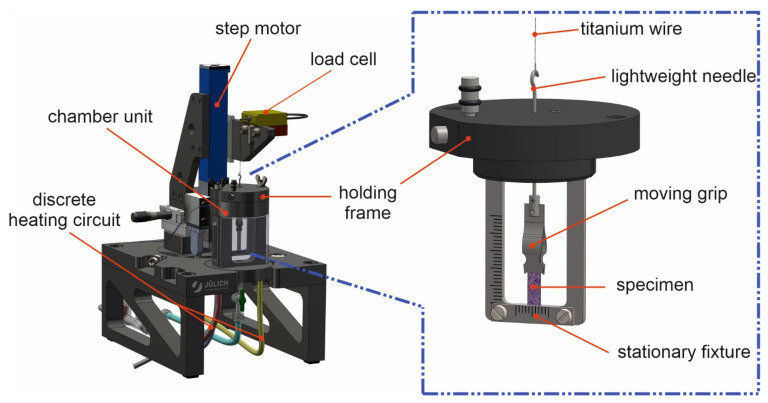
Tissue stretcher setup for tensile measurements. The test specimen is mounted between a stationary fixture and a moving grip that can be locked in position until the holding frame is placed in the chamber unit and the needle is hooked into a titanium wire loop. Afterwards, it is precisely moved by a linear step motor and resulting forces are measured by a load cell. Samples can be kept fully viable in a liquid and temperature-controlled environment during the whole experiment. A front glass plate of the incubation chamber allows low-magnification imaging during experiments.

In this study, a custom-made stretching device for long-term, strain-controlled measurements of vital and even delicate tissue samples was developed and used. Adapted from our previously published stretcher for elastic silicone chambers [29,30], the uniaxial movement is performed by a step motor that drives a linear translation stage (PA 24x29-30-SM02-Sonder, Steinmeyer, Germany). A custom-made LabVIEW-based (Version 2.0 FTDI, LabVIEW 2019, National Instruments, Austin, TX, USA) software tool enables the accurate spatiotemporal control of various parameters as, e.g., stretch frequency, amplitude and time interval between stretch cycles. The stage hosts a force transducer (SI-KG4 + Amplifier BAM21-LC, World Precision Instruments, Sarasota, FL, USA) for sensitive force measurements which can physically be connected to the grip by a titanium wire and a needle (Figure 1). The specimen is mounted in a stationary holding frame on one side and in a fine, 5 mm wide clip made of steel sheet on the other side. During all measurements, the specimen is located in an environmentally controlled and medium-filled incubation chamber. A circulation thermostat (RMS6, Lauda, Lauda-Königshofen, Germany) flows heating liquid through the separated double-walled back of the incubation chamber to maintain a temperature of 37 °C. Additionally, an observation window on the front side allows a permanent visual control of the test specimen during all steps of the experiment.

### 2.2. Tissue Stretcher Calibration

To calibrate the force measurement of our tissue stretcher, cuboidal elastomer samples of the polydimethylsiloxane two-component formulation Sylgard 184 (Dow Chemical, Midland, MI, USA), a previously well-characterized material [30,31], were prepared in certain mixture ratios and the Young’s modulus was determined via successive indentation as already described [32]. The samples were mounted in the apparatus with 10 mm distance between the specimen holders and placed in the buffer-filled chamber unit. A prestretch of 3% (0.3 mm) was applied with an equilibration phase of 30 min. Subsequent straining was performed in two cycles of three successive stretch and release steps with 4% amplitude each (12% in total). Each step was held for 15 min. The dimension of the test samples in unloaded conditions were determined and the sectional area was used for calculation of the tensile stress σ as force per area unit with a Poisson’s ratio of 0.5. In the resulting stress-strain curve, the mean slope of linear regressions yielded the Young’s modulus.

### 2.3. Tissue Stretcher Noise Characterization

After system equilibration for at least 30 min, the holding grip was lowered and raised in buffer without any sample attached. Upper and lower amplitude were held for 1 min each; the full cycle was repeated four times. Force values were sampled at a rate of 10 Hz. For each sequence, histograms of all forces measured in the lower position and in the upper position, respectively, were taken. The cumulative normal distribution was fitted to these histograms. Noise value reported is the mean of all standard deviations from these fits. Amplitudes from these fits were used to measure buoyancy of the grip holder.

### 2.4. Generation of a Simplified Epidermis Equivalent (SEEs) as Multilayered Model System

Murine keratinocytes were isolated from wild type mice and routinely cultured in medium with low Ca^2+^ (50 µM) as described previously [33]. To generate the multilayer epidermal model system, cells were grown on tissue culture plastic until confluence and switched to high Ca^2+^ medium (1.8 mM) to induce cell-cell contact formation. After 18–24 h, Ca^2+^ was depleted with EGTA (5 mM, E3889, Sigma, St. Louis, MO, USA) in PBS for 1 min and cells were trypsinized for 7 min (0.05%, 25300062, Thermo Fisher Scientific, Waltham, MA, USA). Subsequently, cells were replated in high density (4 × 10^5^ keratinocytes per cm^2^) in a total volume of 800 µL on fibronectin (20 µg mL^−1^, 356008, Corning Inc., Corning, NY, USA) coated silicone elastomer chambers (4 cm², [30]) made with the two-component silicone SORTA-Clear 12 (Smooth-On, Macungie, PA, USA) in a 1:1 ratio. After 6 h, dead and non-adhered cells were removed and fresh Ca^2+^-containing medium was added. Resulting multilayer models were incubated overnight at 37 °C and 5% CO_2_.

To transfer the simplified epidermis equivalents (SEEs) as free floating sheets in the tissue stretcher, one sidewall of the chamber was removed and the multilayered sheet was gently detached from the silicone using fine forceps. Subsequently, the SEE was folded 2 times and transferred on a C-shaped PVDF-membrane frame, which served as carrier and distance piece. Together they were mounted in grip and fixture of the holding frame. The lateral ligament of the PVDF frame allowed accurate length maintenance of the specimen. After mounting, the frame was cut and the prepared sample placed in the medium-filled incubation chamber. After connecting the motor-driven load cell with the moving grip, the needle was unblocked for subsequent analysis.

### 2.5. Preparation of Ex Vivo Full-Thickness Back Skin Explants from Fetal and Adult Rats

The tissue used in this study was isolated from animals prepared for other projects (Animal testing license: 81-02.04.2018.A90, LANUV NRW, Germany) and no additional animals were harmed. A pregnant rat (WISTAR, Janvier Labs, Le Genest-Saint-Isle, France) was sacrificed by CO_2_ and decapitated according to local regulations; 18-day-old fetuses were directly removed and decapitated before the full-thickness back skin was carefully excised and placed in cold Hank’s balanced salt solution (1× HBSS, H6648, Sigma, USA). To prevent the tissue from excessive mechanical stress during preparation, a removal of underlying hypodermal tissue was explicitly avoided. The explants were cut into rectangular pieces of 16 × 5 mm with its long axis in cranial-caudal orientation and stored in DMEM/F-12 medium supplemented with HEPES (15 mM, 11039021, Thermo Fisher Scientific, USA), penicillin (100 U mL^−1^), streptomycin (100 µg mL^−1^, 15140122, Thermo Fisher Scientific, USA) and FBS (10%, 10270106, Thermo Fisher Scientific, USA) at 37 °C until further use in stretching experiments within 8 h after isolation. For this, the fetal rat skin explants were transferred on a PVDF-membrane frame and mounted as described for SEEs above.

The adult skin samples were also isolated from the animal’s back. Therefore, a region of approximately 3 × 3 cm next to the spine was depilated and the used dimensions of 5 × 10 mm with long axis in cranial-caudal orientation were marked with waterproof ink. The skin was generously excised and placed in cold HBSS. Subsequently, tissue samples were cut to exact size and stored in medium at 4 °C until further use. All experiments with adult explants were equally performed at 37 °C after placing in the medium-filled chamber unit as described for fetal skin and SEEs. All measurements were started within 24 h after tissue isolation.

### 2.6. Tissue Thickness Determination

Tissue explants and SEEs were fixed in formaldehyde (3.7% in PBS, 1040021000, Sigma, USA) overnight at 4 °C (rat skins) or for 30 min at 37 °C (multilayer models). After brief washing in PBS, the adult rat skin was manually cut into thin slices with a sharp razor blade and placed laterally on a glass slide with some drops of buffer. Images of the slices and a calibrating scale were taken on a binocular microscope (Stemi 508, Zeiss, Jena, Germany) equipped with a camera (EOS 600D, Canon, Tokyo, Japan). The fetal rat skin was transferred in dissolved agarose (2% in PBS, A9539, Sigma, USA) at 60 °C, filled in cryomolds (10 × 10 × 5 mm, 4565, Tissue-Tek, Sakura Finetek, Alphen aan den Rijn, The Netherlands) and cured at room temperature. Subsequently, the agarose blocks were cut in 50 µm slices at the VT1000 S vibratome (Leica Microsystems, Wetzlar, Germany), collected on glass slides and imaged with a light microscope (Axio Vert.A1, Zeiss, Germany). Since the multilayered SEEs were too thin for proper sectioning, the fixed cell sheets were permeabilized with Triton-X 100 in PBS (1%, T8787, Sigma, USA) for 15 min at room temperature, washed with PBS and stained with Phallodin-Atto633 (1:300, 68825, Thermo Fisher Scientific, USA) for 4 h at room temperature. Cell nuclei were stained with NucBlue in PBS (R37606, Thermo Fisher Scientific, USA) for 1 h at room temperature. After washing with PBS, the samples were mounted in Fluoromount and covered with a cover glass. Confocal z-stacks of the full thickness layer were taken at a laser scanning microscope (LSM 880, Zeiss, Germany). Thicknesses were determined as mean of several independent measurements.

### 2.7. Tissue Stretch Protocol and Data Analysis

After transfer of the samples in the tissue stretcher and complete cut of the PVDF transfer frames, the starting sample length was set to 10 mm using a single-lens reflex camera (EOS600D, Canon, Japan) equipped with a macro objective (Macro lens EF-S 60 mm, Canon, Japan). After equilibration for approximately 30 min, the respective protocol was directly started. Data points were recorded every 0.1 s. Photos of the tissues were manually taken during static stretch protocol at certain time points for every cycle. The normalization of the initial force relaxation for each strain cycle was related to each cycle’s peak force. Accordingly, the mean and standard deviation of at least 3 different samples for every data point were calculated. Furthermore, the tissue force recovery was defined as the mean peak force of a certain cycle divided by the mean peak force of the first cycle. In order to apply cyclic tensile forces, we changed the straining protocol to a trapezoidal approximation of a sine wave for some experiments. Here, the tissues were cyclically loaded and unloaded with identical velocity compared to static stretch. The dwell time at loaded and unloaded position was set to 0.4 s to result in a stretch frequency of 0.3 Hz. For further analysis and visualization, only peak forces of every cycle were plotted.

### 2.8. Viability Assays of Fetal Rat Skin and SEEs

For viability analysis, fetal rat skin explants were stretched and subsequently transferred in medium with fluorescein diacetate (25 µg mL^−1^, F7378, Sigma, USA) and ethidium bromide (10 µg mL^−1^) for 15 min at 37 °C. A positive control was kept in medium supplemented with 1% sodium azide for 2 h prior to staining. Imaging was directly performed after a brief washing step with fresh medium at the LSM 880 confocal microscope (Zeiss, Germany) with a 20× plan apochromatic objective and appropriate filter settings. The SEEs were transferred in trypsin-EDTA solution (0.05%, 25300062, Thermo Fisher Scientific, USA) and incubated for 10 min. Once the cell sheets started to break into pieces by smooth agitation, the SEEs were mechanically dissolved by pipetting up and down. The cells were then centrifuged and the pellet either resuspended in PBS with FBS (10%, 10270106, Thermo Fisher Scientific, USA) or in PBS with EtOH (35%) as positive control. The following viability assay was performed with the LIVE/DEAD™ Fixable Cell Stain Kit (L34963, Thermo Fisher Scientific, USA) according to the manufacturer’s protocol and analyzed by flow cytometry (Cytoflex S, Beckman Coulter, Brea, CA, USA).

### 2.9. Data Analysis

Statistical analysis and plots of recorded data were performed using GraphPad Prism software (Version 9, GraphPad Software Inc., San Diego, CA, USA). Microscopic images of tissue sections were evaluated using Fiji ImageJ software (Version 1.53c, open source scientific image processing [34]). Schemes and figures were designed using CorelDRAW software (Version 21, Corel, Ottawa, ON, Canada).

## 3. Results

### 3.1. Experimental Setup Development for Sensitive Tensile Measurements of Living Samples

In order to enable precise and sensitive tensile test measurements in a cell culture environment, we developed a new tensile test instrument for long-term, strain-controlled experiments (Figure 1). Here, the specimen is mounted in a removable holding frame and placed in an environmentally controlled chamber unit. This unit can be filled with approximately 30 mL medium or buffer and run in batch or continuous flow mode. The discrete heating unit enables temperature control of the sample microenvironment and drives a convective flow of nutrients. A linear translation stage driven by a step motor moves a platform that is attached to the moving grip with preselected velocity, amplitude and dwell times. A load cell can be interconnected and enables real-time measurements of applied tensile forces. Control of the linear translation stage and readout of the load cell are performed by a custom-designed computer program.

**Figure 2:**
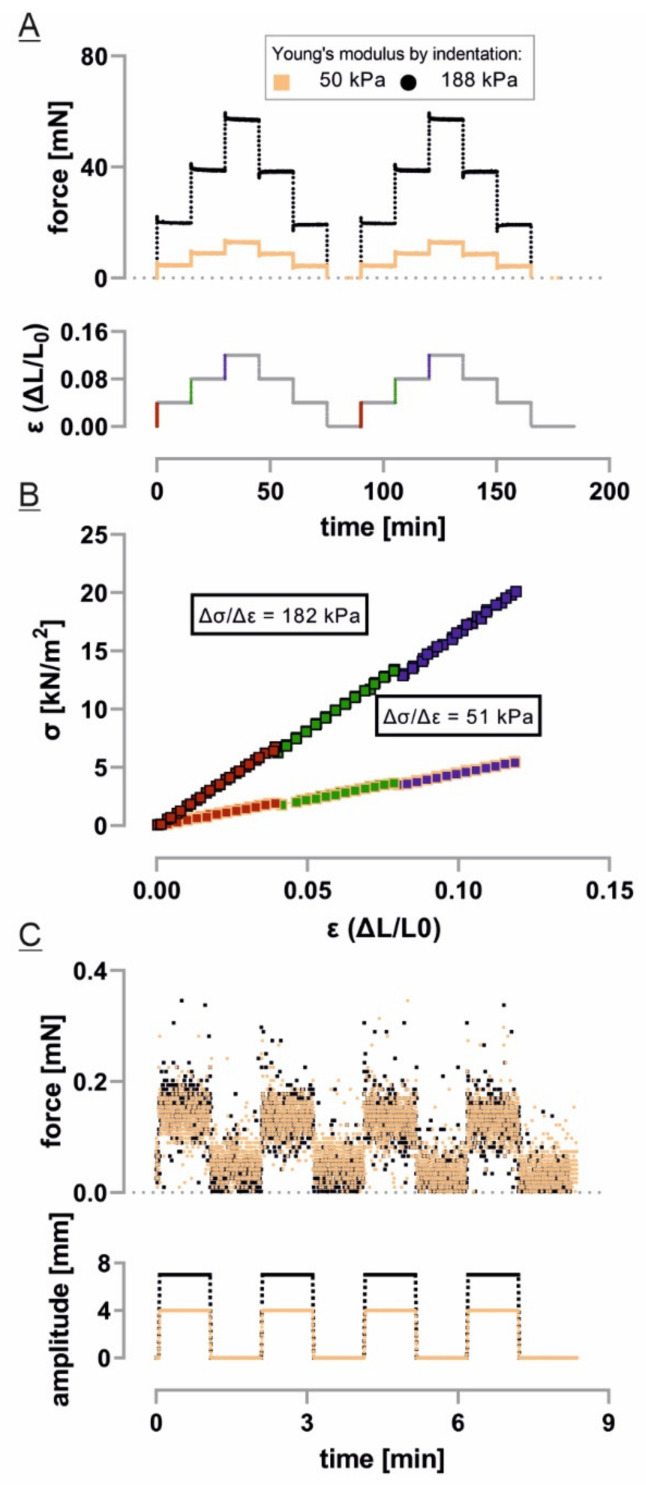
Setup calibration: (**A**) Two different elastomer samples of defined geometry and known Young’s modulus were strained stepwise from 0–0.04, 0.04–0.08 and 0.08–0.12. The resulting forces were measured with a rate of 10 Hz; (**B**) Taking the sample’s dimensions into consideration, the linear correlation of stress and strain enables the calculation of expected Young’s moduli for every step (indicated in red, green and blue, respectively, in (**A**) and (**B**)). Stated here is the mean Young’s modulus of all steps; (**C**) The sensitivity of the setup was shown by measuring the very small buoyancy correction to the force. To this end, the instrument’s needle was raised in liquid without a sample mounted to the amplitudes used in our experiments.

We characterized the predicted high temporal resolution and sensitivity for force measurements with elastomer test specimens of known, well-defined Young’s moduli. The stepwise straining of such elastomer samples resulted in a linear force increase, followed by a fast stress relaxation that approached a plateau within minutes (Figure 2A). For repetitive cycles of stepwise straining, almost equal force levels of equivalent steps were reached as expected for viscoelastic rubbers in their linear regime. Moreover, linear fitting of stress and strain data as described in the Materials and Methods section yielded Young’s moduli very similar (deviation ≤ 3.3%) to the values determined by established indentation measurements (Figure 2B). Ultimate sensitivity was assayed by measuring the buoyancy of the connector needle linked to the moving grip. To this end, the needle was moved up and down by 4 mm or 7 mm, respectively, in the liquid. This led to a small, measured force increase of about 70 µN at 4 mm amplitude and about 90 µN at 7 mm amplitude, respectively. As forces on real samples were much higher, buoyancy was not corrected for. At these amplitudes, buoyancy forces are slightly larger than the noise level of 22 µN at a sampling frequency of 10 Hz (Figure 2C). The average drift of force values during the 8 min intervals of these measurements was 1.6 µN min^−1^.

### 3.2. Tested Tissues Differ in Complexity, Maturation and Composition

**Figure 3:**
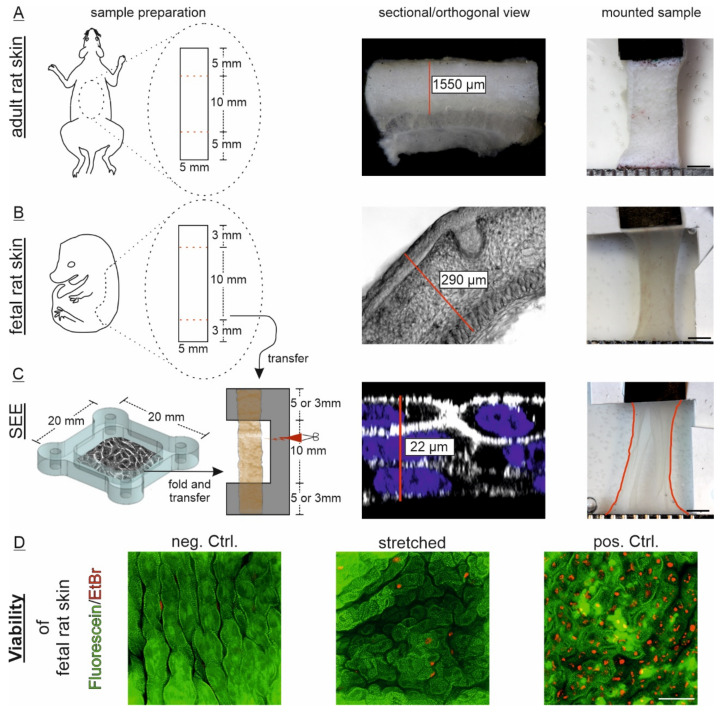
Skin model systems to analyze dermal and epidermal mechanical properties. Three models of different maturation state and composition were comparably prepared: (**A**) Adult rat skin explants were excised from back skin and the test dimension used for analysis previously marked in situ. The mean dermo-epidermal thickness (exemplarily labeled with red line) was manually determined from tissue slices (*n* = 16). Underlying hypodermal tissue was omitted here; (**B**) Back skin from embryonic day 18 rats was cut into indicated test dimensions after isolation. Analysis length was set to 10 mm initial length using a C-shaped PVDF frame (gray). Mean tissue thickness was measured from 14 independent sections (*n* = 14); (**C**) Similar PVDF frames were used for transfer of multilayered, simplified epidermis equivalents (SEEs). Therefore, the 20 × 20 mm cell sheets were folded 2 times along one axis to obtain the 5 × 20 mm test dimension. The mean cell sheet thickness is indicated (*n* = 17); (**A**–**C**) Right images show mounted samples previous to start of the test protocol. The lateral white PVDF transfer frame in fetal skin and SEE samples were cut after mounting and had therefore no influence on subsequent force measurements. Scale: 2 mm. Red label illustrates the outline of SEE; (**D**) Viability assay of epidermal cells via immunofluorescence was performed with fetal rat skin after transfer and stretching for at least 4 h. Non-transferred but identically prepared samples served as controls. Fetal skin in positive controls were deliberately killed with azide. Scale: 50 µm.

In this study, epidermal model systems of different complexity, structure and composition were mechanically characterized and compared. The full-thickness back skin of adult rats is composed of a fully developed and sparsely cellularized dermal layer with attached hypodermal fatty tissue and a considerably thinner epidermal layer. Since the initial and non-preconditioned mechanical response should be measured in this study, mechanical stress was avoided as much as possible and adipose tissue therefore not entirely removed. We also performed some tensile tests on skin in which the subcutaneous tissue was previously separated and did not find major differences in curve progression and force levels. For this reason, we used the dermo-epidermal thickness for tissue stress estimation and neglected the attached fatty layer. The mean dermo-epidermal thickness was determined to be around 1550 µm (SD 130 µm) with the cornified epidermis of approximately 50 µm (Figure 3A). Even though it lacks a cornified layer, the epidermis of rat embryos exhibited approximately the same thickness as the one of adult rats. However, the highly cellularized, immature dermal layer was much thinner and the hypodermal tissue was only loosely attached and scarcely present in tissue sections (Figure 3B). Overall, the mean dermo-epidermal thickness of 290 µm (SD 36 µm) was less than 20% of adult rat skin. To analyze the impact of the dermal ECM on strength and elasticity, we additionally developed a simplified epidermis equivalent (SEE) based on cultured murine keratinocytes. This system could be mounted in our tensile test instrument (Figure 3C). Here, the keratinocytes were exclusively connected by intercellular junctions in a cell sheet of 3–5 cell layers adding up to a mean thickness of 22 µm (SD 3.3 µm).

### 3.3. Static Stretch Shows Viscoelastic and Plastic Properties and Indicates Partial Recovery

**Figure 4:**
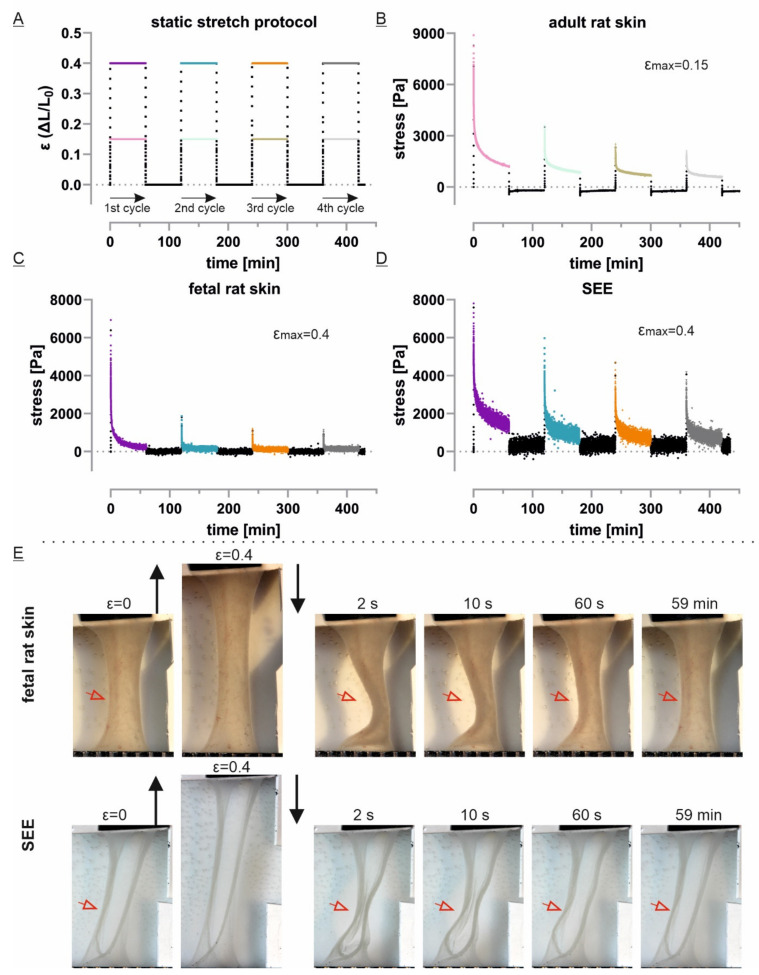
Repetitive static stretch protocol uncovers viscoelastic properties and tissue strain recovery: (**A**) The test protocol provides four cycles of 1 h at strain position followed by 1 h at zero position; (**B**–**D**) The stress curves of one representative experiment for each model system are shown. Tissue stress was calculated from the estimated cross-sectional area after isolation and fixation (see Figure 3). Data was recorded every 0.1 s; (**E**) Images on the right were taken at indicated time points after moving back to zero position. The tissue dimension before and during stretch are indicated on the left. The visible bending and length recovery (indicated by red arrowheads) was reproducibly observed over several samples and all repetitive cycles.

To characterize the mechanical properties of all skin models, we used a static stretch protocol of four repetitive cycles of 1 h strain followed by 1 h rest at zero position (Figure 4A). Here, we chose to use a strain level of 15% for adult skin instead of 40% for the other models in order to reach similar tissue peak stress levels after the initial ramp stretch between the different models. Moreover, the measured tissue stresses were below stress levels that were previously reported to induce fibrillary orientation in the adult epidermis [9,11,21]. Indeed, even though the absolute force levels differ between 15% and 40% for adult rat skin explants, we did not find major differences in curve progression and normalized force relaxation behavior in our experimental setup (Figure S2B,C). Furthermore, viability assays confirmed full biocompatibility of our experimental setup for fetal rat skin epidermis and multilayered SEEs (Figure 3D, Figure S1). All three epidermal model systems reached their maximal stress immediately upon initial first strain followed by a stress relaxation phase (Figure 4B–D). The stress relaxation could be divided into a first short phase of fast stress relaxation followed by a much slower phase. In contrast to fetal rat skin, SEEs as well as adult rat skin did not reach stable stress values within 1 h of static strain. Furthermore, the stress values immediately dropped to basal levels after returning to zero strain position in all experiments.

Interestingly, strained tissue also recovered to its initial length during the following rest period with fastest response during the first seconds (Figure 4E). This could be monitored through the frontal observation window by time-resolved manual imaging. Length recovery was best visible for fetal rat skins and SEEs and was reproducibly observed for all repetitive cycles independent of the cycle’s peak force. However, even brief tissue denaturation resulted in loss of tissue length recovery (Figure S3).

### 3.4. Only Adult Skin Differs in Relaxation Behavior between Initial and Repetitive Cycles

**Figure 5:**
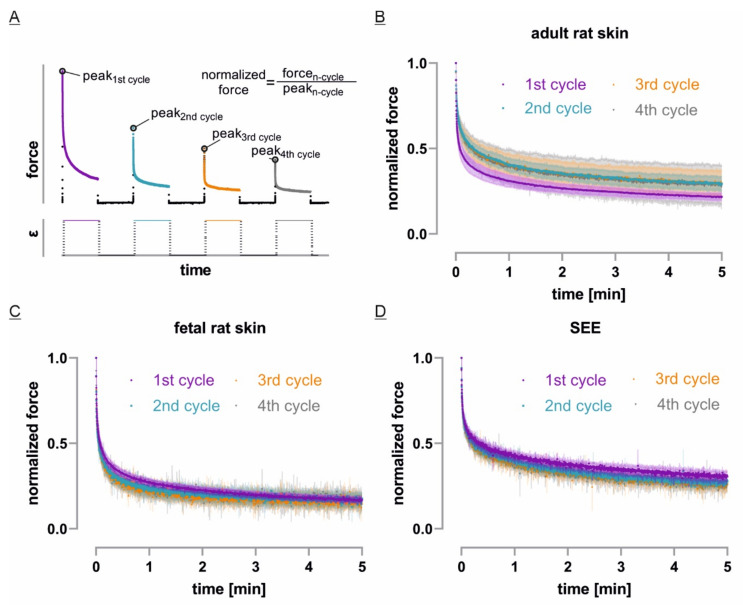
Tissue stress relaxation during repetitive static stretch and rest cycles: (**A**) To compare tissue behavior for repetitive cycles, force values of every stretch cycle were normalized to its initial peak force; (**B**–**D**) The initial stress relaxation curves for each tissue system were plotted as mean in dark color and SD in bright color over time (fetal: *n* = 3, adult and SEE: *n* = 4).

To evaluate the influence of repetitive cycles on the force relaxation behavior of our different model systems, we normalized each stretch cycle to its initial peak force (Figure 5A). Surprisingly, the fetal rat skin explants showed the fastest force decay of all tested systems and no difference between repetitive stretch cycles (Figure 5C). Even though the SEEs relaxed slower and declined to approx. 28% of the peak force value after 5 min compared to only approx. 17% in case of fetal rat skin, SEEs also exhibited no difference in relaxation behavior between repetitive stretch cycles (Figure 5D). In contrast, the first cycle of adult rat skin showed a significantly faster force relaxation than following cycles, which then exhibited no further difference. In mature skin samples, force decreased to approx. 30% of its initial value after 5 min in load position (Figure 5B).

### 3.5. ECM-Free Epidermis Equivalents Recover Like Previously Stretched Tissue

**Figure 6:**
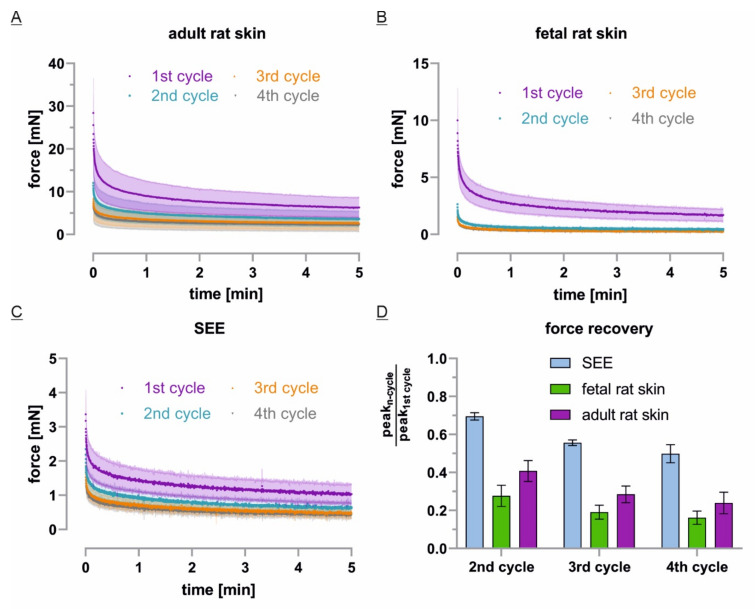
Tissue force recovery upon repetitive static stretch and rest cycles: (**A**–**C**) The initial force decay for each tissue system was plotted as mean in dark color and SD in bright color at relative time scale (fetal: *n* = 3, adult and SEE: *n* = 4); (**D**) The tissue force recovery was calculated as mean and SD of the cycle’s peak force divided by the highest, first peak force value.

The absolute force decay of repetitive static stretch cycles revealed further similarities and differences between the mechanical properties of the tested tissues. Here, fetal rat skin showed not only the most striking force decrease under stretch but also the lowest force recovery after a 1 h rest period (Figure 6B,D). Especially in the 3rd and 4th cycle, measured forces dropped within minutes to values that were only slightly higher than the expected force increase of buoyancy (e.g., 0.27 mN, SD 0.03 mN, and 0.25 mN, SD 0.07 mN after 5 min). Even though stretch increased force in adult rat skin to values that were almost three times the initial force of fetal rat skin, after normalization by peak force, similar curves were obtained during the first stretch (Figure 6A,B; Figure S4). However, the overall force recovery after rest was about 10% higher for adult compared to fetal skin. Interestingly, the highest force recovery relative to the first peak were found for SEEs in all cycles (Figure 6D). Here, the tissue reached 70% (2nd cycle), 56% (3rd cycle) and 50% (4th cycle) of the initial peak force, respectively. Nevertheless, the relative force decay of repetitive static stretch cycles was very similar to adult rat skin during stretch cycle 2–4 (Figure 6C; Figure S4). Intriguingly, when force values were compared with the previous static stretch cycle, all systems reached 70–80% (3rd cycle) and 80–90% (4th cycle) after 1 h rest. This less pronounced difference of repeatedly stretched tissues indicates that the model systems potentially achieve a mechanical steady state in their particular condition.

### 3.6. Fast Cyclic Strain Has a Tissue Dependent Impact on Force Relaxation

**Figure 7:**
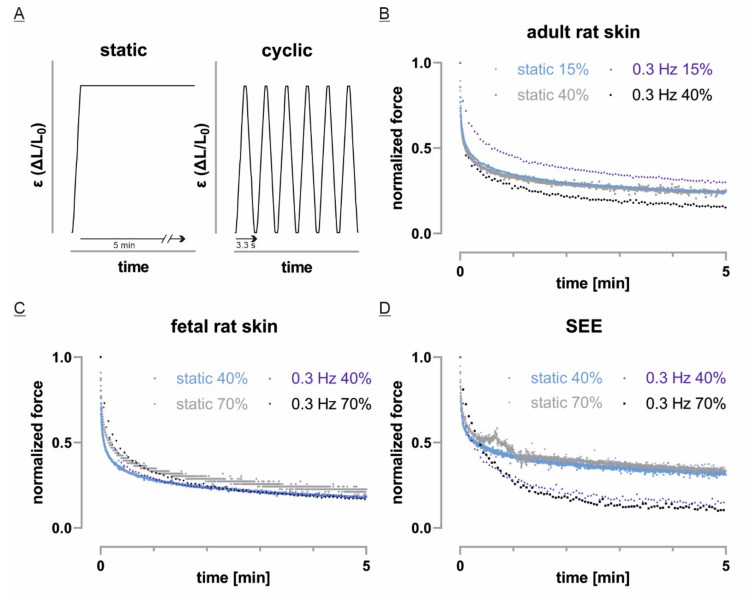
Static and cyclic strain affects tissues differently: (**A**) The different tissue models were subjected to either static stretch with a constant load or trapezoid-cyclic stretch with 0.3 Hz but equal strain velocity and amplitude; (**B**–**D**) Normalized force relaxation curves of individual experiments were plotted. Note that both stretch protocols were performed at two different amplitudes. For better visualization, only peak forces of cyclic stretch experiments are shown.

To gain a deeper understanding of mechanical properties, we additionally applied cyclic stretch and higher strain amplitudes to subsequently analyze force relaxation (Figure 7A). In case of fetal rat skin, neither higher strain levels nor cyclic straining with 0.3 Hz changed the force relaxation rate significantly (Figure 7C). Adult rat skin generally showed a higher variance in the different stretch protocols (Figure 7B). Furthermore, static strain potentially resulted in a slightly faster force relaxation compared to slower relaxation when stretch was cyclically applied. Nevertheless, adult skin also showed similar force relaxation curves in repeated measurements independent of straining type and amplitude (Figure 7B). Besides, a strain amplitude independent relaxation behavior could also be observed for SEEs. However, a clear difference was found when equivalents were stretched cyclically. Here, measured peak forces decreased within minutes to less than half of related values for static stretch experiments (Figure 7D) and ranged even slightly below corresponding curves of fetal skin.

## 4. Discussion

Due to their inherent fragility and often low reaction forces, biomechanical testing of delicate tissue samples or cell culture tissue models under physiological conditions can be quite challenging. The instrument developed here closes the gap between classic tensile test devices known from material sciences and very sensitive experimental approaches to measure mechanical properties on a molecular or cellular level [35,36,37].

Our instrument exhibited a noise level of 22 µN at a sampling rate of 10 Hz. This noise in forces corresponds for simplified skin equivalents to a standard deviation in stresses of 100 Pa; for fetal skin, the corresponding value is 7.6 Pa. As can be seen, for example, in Figure 4, this level of scatter is by far sufficient for tensile tests at physiologically relevant amplitudes. In many instances, relevant data can be averaged of much longer periods than 0.1 s. Therefore, most parameters can be measured with significantly higher accuracy. Moreover, many mechanical experiments on very soft tissues suffer from artifacts caused by clamping-induced cutting of the tissue. These we could circumvent for the softest samples by the use of a relatively stiff carrier made of PVDF membrane sheets. In addition, the frames facilitate a reproducible free tissue length at zero strain position based on their physiological length in situ. The lateral ligament of the PVDF transfer frames was cut after mounting of the sample and did, therefore, not affect the measurement. As a result, we never observed significant artifacts due to slipping or tearing of tissue at the fixtures.

In this study, the device enabled, for the first time, a direct comparison of mature full-thickness skin, fetal skin and an ECM-free epidermal model tissue (simplified epidermis equivalent—SEE) (Figure 3 and Figure 4). Please note that under physiological or growth conditions, fetal skin and SEE are immersed in medium whereas the dermal side of adult skin is in contact with interstitial fluid while the epidermal side is exposed to air. Therefore, our aim of a direct comparison of adult skin, fetal skin, and epidermal equivalents necessitated an instrument design where samples are immersed in medium throughout the measurements. However, for adult skin alone, measurement in air and in medium are equally close to physiology. As measurement in air is much easier and can be performed with commercial equipment, most work on mature skin is done in (humidified) air. Nevertheless, especially in the low strain region, fluid contact and detailed nature of the fluid are important determinants for the mechanics of soft collagenous materials, in general, and skin, in detail [38,39,40]. All mechanical analyses in our study were performed with hydrated tissue in a medium-filled chamber. Thus, it is not surprising that we measured tissue stresses on adult skin that are lower than most reported values at identical nominal strain.

Another important aspect in which our measurements on adult skin are set apart from the bulk of the literature is the strain regime. Whereas most measurements on adult skin were performed in the high strain linear regime, where collagen fibrils are almost uniformly aligned and intra- and interfibrillary sliding are the most important mechanisms of elasticity [5,10,11], we confined our measurements to lower strains and, as a consequence, significantly lower stresses (Figure S2A). Additionally, we were interested in both the initial, first response to mechanical stretch but also the history-dependent behavior of repeated stretch and release cycles. In accord with this choice, we did not precondition our samples as it is routinely done for measurements that focus on material response in the linear regime [6].

In our experiments, we focused on physiological conditions and therefore stress levels below 30 kPa, which were largely ignored in previous studies (Figure 4A–D). In fact, a recent, careful study of skin microstructure during stretch reported collagen fiber reorientation, stretching and sliding to be triggered only by stress above 1 MPa (corresponding to strains of more than 40%) whereas at lower deformations, straightening of the curved collagen fibers prevailed [11].

As our samples were not preconditioned, the first force relaxation curve of adult skin differed from later ones (Figure 5B, [5]). Although not fully understood, this history-dependent change in viscoelasticity is generally explained and modeled by a microstructural adaptation of ground substance and elastin fibers at low strain and a time-dependent increase in reference length of the collagen fibers at enhanced strain amplitudes [41]. Our experiments confirm that this effect is still present after 1 h in rest position and supports the idea of a robust microstructural strain-dependent adaptation of dermal tissue. Furthermore, neither fetal rat skin nor SEEs showed a similar hysteresis (Figure 5C,D). While our epidermal model system does not contain any ECM fibers, these mechanically relevant proteins are not yet entirely secreted and cross-linked in the highly cellularized fetal rat skin dermis [15]. This immature condition of embryonic day 18 rat skin led to a markedly lower viscoelastic phenotype in previous shear creep tests and was surprisingly well modeled by soft gelatin gels [42]. The gel-like characteristic was not only displayed by a notably fast force relaxation in repeated stretch cycles but also by a very low force recovery during 1 h rest at starting position (Figure 5C and Figure 6B,D). Besides, earlier histological studies have demonstrated severe fractures in the epidermis and dermis of fetal skin after creep tests until failure [43], which argues for plastic deformations or, in other words, irreversible change of dermal microstructure. Based on well-established analytic models of poroelasticity [38] and current simulation models [44], the remaining small initial peaks in repetitive stretch cycles, followed by an almost instantaneous drop to basal force values (Figure 4C), possibly reflect hydrodynamically driven water extrusion in an ultrasoft, poroelastic material.

Interestingly, tissue length is not equally affected and revealed an impressive recovery. Indeed, fetal skin samples reproducibly regain their initial length within the rest periods of the test protocol (Figure 4E). This tissue strain recovery was similarly observed for epidermal equivalents in our experiments. Together with the absence of measurable equilibrium forces in fetal skin and the total absence of ECM itself in SEE, these observations implied an ECM-independent process. Although limitations in profile observations during tensile testing caused by the incubation chamber design prevented own verification, earlier studies have already demonstrated that the tissue dimension of rabbit skin was restored ex vivo after a long relaxation time [45] but full recovery of mechanical properties was exclusively found in vivo after several hours of rest [18]. Upon length reduction, suspended cell mono- and multilayers have previously shown an actomyosin-dependent mechanical adaption that results in fast tissue flattening and length recovery [46]. This active process is dominated by structural changes in the actomyosin cortex and is responsible for stress relaxation and a change in tissue length [47]. In accordance with this concept, keratinocytes of fetal epidermis or our epidermal model system actively restored their initial dimension while dead tissues were unable to regain their pre-stretch configurations (Figure 3D; Figure S3). Additionally, our SEEs displayed a high force recovery after a first stretch while mature skin showed a markedly larger decrease of tissue peak force (Figure 6D). Interestingly, in the second half of the stretch protocol, both model systems recovered at a similar rate to the previous cycle. While during the first stretch, a formerly unstressed adult rat skin behaves more like a gel-like material similar to fetal skin, the normalized force relaxation of the following cycles is almost identical to the relaxation of ECM-free SEEs (Figure S4). Although structurally simplified, we therefore conclude that in physiological regimes of repetitive force application, our multilayered SEEs mimic dermal mechanics and possess many features of full-thickness skin.

In the response to applied forces, a fundamental difference between mature connective tissue and living cells emerged when we applied comparatively fast cyclic stretch instead of a constant strain. While in SEEs, higher amplitudes did not affect force relaxation, we observed a surprisingly faster relaxation when the specimens were cyclically instead of statically stretched (Figure 7D). However, cyclic stretch did not generally alter the relaxation behavior as shown for adult full-thickness skin in our observations here and earlier studies by others [48]. On a subcellular scale, all cytoskeletal systems show a cyclic strain-induced reorientation in a specific time-dependent manner [49]. In particular, in mechanically integrated cell monolayers, F-actin reorientation upon stretch goes along with cooperative reinforcement that is strongly dependent on intercellular junctions [29]. Cytoskeletal deformations and subsequent dynamical regulation of actomyosin were identified to be responsible for structural integrity and stress relaxation of cell monolayers [47,50]. When cyclic stretch is applied, the cytoskeleton fluidizes after a few minutes, followed by a substantial gain in order with increasing duration of cyclic straining [51]. Consequently, we propose similar strain-induced adaptation processes in native epidermal tissue under physiological conditions.

In summary, we present here a direct mechanical comparison of simplified epidermal equivalents, fetal, and adult rat skin. Despite the structural differences between these samples, mechanical behavior exhibited surprising similarities that highlight the high value of epidermal equivalents as in vitro model systems for basic research in dermatology and epidermal cell biology.

## Data Availability

All datasets generated during and/or analyzed during the current study are available from the corresponding author on reasonable request.

## Supplementary Materials

The following are available online at https://www.mdpi.com/article/10.3390/cells10071834/s1, Figure S1: SEEs are still viable after long-term static stretch experiments, Figure S2: Adult skin responds similar to 15% and 40% of static stretch, Figure S3: Tissue strain recovery is an active process, Figure S4: Multilayered SEEs mimic adult rat skin after preconditioning in first stretch cycle.

## References

[1] Wong V.W., Akaishi S., Longaker M.T., Gurtner G.C. (2011). Pushing back: Wound mechanotransduction in repair and regeneration. J. Investig. Dermatol..

[2] Simpson C.L., Patel D.M., Green K.J. (2011). Deconstructing the skin: Cytoarchitectural determinants of epidermal morphogenesis. Nat. Rev. Mol. Cell Biol..

[3] Wei J.C.J., Edwards G.A., Martin D.J., Huang H., Crichton M.L., Kendall M.A.F. (2017). Allometric scaling of skin thickness, elasticity, viscoelasticity to mass for micro-medical device translation: From mice, rats, rabbits, pigs to humans. Sci. Rep..

[4] Fenske N.A., Lober C.W. (1986). Structural and functional changes of normal aging skin. J. Am. Acad. Dermatol..

[5] Fung Y.C. (1993). Biomechanics: Mechanical Properties of Living Tissues.

[6] Joodaki H., Panzer M.B. (2018). Skin mechanical properties and modeling: A review. Proc. Inst. Mech. Eng. Part H.

[7] Liu Z., Yeung K. (2008). The preconditioning and stress relaxation of skin tissue. J. Biomed. Eng..

[8] Ottenio M., Tran D., Ni Annaidh A., Gilchrist M.D., Bruyere K. (2015). Strain rate and anisotropy effects on the tensile failure characteristics of human skin. J. Mech. Behav. Biomed. Mater..

[9] Wong W.L., Joyce T.J., Goh K.L. (2016). Resolving the viscoelasticity and anisotropy dependence of the mechanical properties of skin from a porcine model. Biomech. Model. Mechanobiol..

[10] Ní Annaidh A., Bruyère K., Destrade M., Gilchrist M.D., Otténio M. (2012). Characterization of the anisotropic mechanical properties of excised human skin. J. Mech. Behav. Biomed. Mater..

[11] Yang W., Sherman V.R., Gludovatz B., Schaible E., Stewart P., Ritchie R.O., Meyers M.A. (2015). On the tear resistance of skin. Nat. Commun..

[12] Ni Annaidh A., Bruyere K., Destrade M., Gilchrist M.D., Maurini C., Ottenio M., Saccomandi G. (2012). Automated estimation of collagen fibre dispersion in the dermis and its contribution to the anisotropic behaviour of skin. Ann. Biomed. Eng..

[13] Purslow P.P., Wess T.J., Hukins D.W. (1998). Collagen orientation and molecular spacing during creep and stress-relaxation in soft connective tissues. J. Exp. Biol..

[14] Eshel H., Lanir Y. (2001). Effects of strain level and proteoglycan depletion on preconditioning and viscoelastic responses of rat dorsal skin. Ann. Biomed. Eng..

[15] Coolen N.A., Schouten K.C., Middelkoop E., Ulrich M.M. (2010). Comparison between human fetal and adult skin. Arch. Dermatol. Res..

[16] Chin M.S., Lancerotto L., Helm D.L., Dastouri P., Prsa M.J., Ottensmeyer M., Akaishi S., Orgill D.P., Ogawa R. (2009). Analysis of neuropeptides in stretched skin. Plast. Reconstr. Surg..

[17] Kumaraswamy N., Khatam H., Reece G.P., Fingeret M.C., Markey M.K., Ravi-Chandar K. (2017). Mechanical response of human female breast skin under uniaxial stretching. J. Mech. Behav. Biomed. Mater..

[18] Vogel H.G., Denkel K. (1985). In vivo recovery of mechanical properties in rat skin after repeated strain. Arch. Dermatol. Res..

[19] Kang G., Wu X. (2011). Ratchetting of porcine skin under uniaxial cyclic loading. J. Mech. Behav. Biomed. Mater..

[20] Munoz M.J., Bea J.A., Rodriguez J.F., Ochoa I., Grasa J., del Palomar A.P., Zaragoza P., Osta R., Doblare M. (2008). An experimental study of the mouse skin behaviour: Damage and inelastic aspects. J. Biomech..

[21] Afsar-Kazerooni N., Srinivasa A.R., Criscione J.C. (2020). Experimental Investigation of the Inelastic Response of Pig and Rat Skin Under Uniaxial Cyclic Mechanical Loading. Exp. Mech..

[22] Jemec G.B., Serup J. (1990). Epidermal hydration and skin mechanics. The relationship between electrical capacitance and the mechanical properties of human skin in vivo. Acta Derm. Venereol..

[23] Hsu C.K., Lin H.H., Harn H.I., Hughes M.W., Tang M.J., Yang C.C. (2018). Mechanical forces in skin disorders. J. Dermatol. Sci..

[24] Werner N.S., Windoffer R., Strnad P., Grund C., Leube R.E., Magin T.M. (2004). Epidermolysis bullosa simplex-type mutations alter the dynamics of the keratin cytoskeleton and reveal a contribution of actin to the transport of keratin subunits. Mol. Biol. Cell.

[25] Cavanaugh K.J., Oswari J., Margulies S.S. (2001). Role of stretch on tight junction structure in alveolar epithelial cells. Am. J. Respir. Cell Mol. Biol..

[26] Rodrigues Neves C., Gibbs S. (2018). Progress on Reconstructed Human Skin Models for Allergy Research and Identifying Contact Sensitizers. Curr. Top. Microbiol. Immunol..

[27] Frankart A., Malaisse J., De Vuyst E., Minner F., de Rouvroit C.L., Poumay Y. (2012). Epidermal morphogenesis during progressive in vitro 3D reconstruction at the air-liquid interface. Exp. Dermatol..

[28] Monemian Esfahani A., Rosenbohm J., Reddy K., Jin X., Bouzid T., Riehl B., Kim E., Lim J.Y., Yang R. (2019). Tissue Regeneration from Mechanical Stretching of Cell-Cell Adhesion. Tissue Eng. Part C Methods.

[29] Noethel B., Ramms L., Dreissen G., Hoffmann M., Springer R., Rubsam M., Ziegler W.H., Niessen C.M., Merkel R., Hoffmann B. (2018). Transition of responsive mechanosensitive elements from focal adhesions to adherens junctions on epithelial differentiation. Mol. Biol. Cell.

[30] Faust U., Hampe N., Rubner W., Kirchgessner N., Safran S., Hoffmann B., Merkel R. (2011). Cyclic stress at mHz frequencies aligns fibroblasts in direction of zero strain. PLoS ONE.

[31] Merkel R., Kirchgessner N., Cesa C.M., Hoffmann B. (2007). Cell force microscopy on elastic layers of finite thickness. Biophys. J..

[32] Ulbricht A., Eppler F.J., Tapia V.E., van der Ven P.F., Hampe N., Hersch N., Vakeel P., Stadel D., Haas A., Saftig P. (2013). Cellular mechanotransduction relies on tension-induced and chaperone-assisted autophagy. Curr. Biol..

[33] Rubsam M., Mertz A.F., Kubo A., Marg S., Jungst C., Goranci-Buzhala G., Schauss A.C., Horsley V., Dufresne E.R., Moser M. (2017). E-cadherin integrates mechanotransduction and EGFR signaling to control junctional tissue polarization and tight junction positioning. Nat. Commun..

[34] Schindelin J., Arganda-Carreras I., Frise E., Kaynig V., Longair M., Pietzsch T., Preibisch S., Rueden C., Saalfeld S., Schmid B. (2012). Fiji: An open-source platform for biological-image analysis. Nat Methods.

[35] Yap A.S., Duszyc K., Viasnoff V. (2018). Mechanosensing and Mechanotransduction at Cell-Cell Junctions. Cold Spring Harb. Perspect. Biol..

[36] Pinheiro D., Bellaiche Y. (2018). Mechanical Force-Driven Adherens Junction Remodeling and Epithelial Dynamics. Dev. Cell.

[37] Griffin M., Premakumar Y., Seifalian A., Butler P.E., Szarko M. (2016). Biomechanical Characterization of Human Soft Tissues Using Indentation and Tensile Testing. J. Vis. Exp..

[38] Cowin S.C., Doty S.B. (2007). SpringerLink. Tissue Mechanics.

[39] Ehret A.E., Bircher K., Stracuzzi A., Marina V., Zundel M., Mazza E. (2017). Inverse poroelasticity as a fundamental mechanism in biomechanics and mechanobiology. Nat. Commun..

[40] Bircher K., Zundel M., Pensalfini M., Ehret A.E., Mazza E. (2019). Tear resistance of soft collagenous tissues. Nat. Commun..

[41] Lokshin O., Lanir Y. (2009). Viscoelasticity and preconditioning of rat skin under uniaxial stretch: Microstructural constitutive characterization. J. Biomech. Eng..

[42] Harada K., Enosawa S., Zhang B., Yuan W., Chiba T., Fujie M.G. (2011). Evaluation of fetal tissue viscoelastic characteristics for robotic fetal surgery. Int. J. Comput. Assist. Radiol. Surg..

[43] Tsubouchi K., Enosawa S., Harada K., Okamoto J., Fujie M.G., Chiba T. (2006). Evaluation of the relationship between the viscoelastic stress and strain of fetal rat skin as a guide for designing the structure and dynamic performance of a manipulator for fetal surgery. Surg. Today.

[44] Moeendarbary E., Valon L., Fritzsche M., Harris A.R., Moulding D.A., Thrasher A.J., Stride E., Mahadevan L., Charras G.T. (2013). The cytoplasm of living cells behaves as a poroelastic material. Nat. Mater..

[45] Lanir Y., Fung Y.C. (1974). Two-dimensional mechanical properties of rabbit skin. II. Experimental results. J. Biomech..

[46] Wyatt T.P.J., Fouchard J., Lisica A., Khalilgharibi N., Baum B., Recho P., Kabla A.J., Charras G.T. (2020). Actomyosin controls planarity and folding of epithelia in response to compression. Nat. Mater..

[47] Khalilgharibi N., Fouchard J., Asadipour N., Barrientos R., Duda M., Bonfanti A., Yonis A., Harris A., Mosaffa P., Fujita Y. (2019). Stress relaxation in epithelial monolayers is controlled by the actomyosin cortex. Nat. Phys..

[48] Remache D., Caliez M., Gratton M., Dos Santos S. (2018). The effects of cyclic tensile and stress-relaxation tests on porcine skin. J. Mech. Behav. Biomed. Mater..

[49] Zielinski A., Linnartz C., Pleschka C., Dreissen G., Springer R., Merkel R., Hoffmann B. (2018). Reorientation dynamics and structural interdependencies of actin, microtubules and intermediate filaments upon cyclic stretch application. Cytoskeleton.

[50] Harris A.R., Peter L., Bellis J., Baum B., Kabla A.J., Charras G.T. (2012). Characterizing the mechanics of cultured cell monolayers. Proc. Natl. Acad. Sci. USA.

[51] Springer R., Zielinski A., Pleschka C., Hoffmann B., Merkel R. (2019). Unbiased pattern analysis reveals highly diverse responses of cytoskeletal systems to cyclic straining. PLoS ONE.

