# Supplementary material for: Skin under Strain: From Epithelial Model Tissues to Adult Epithelia
Source: Cells. 2021 Jul 20;10(7):1834. doi: 10.3390/cells10071834 (PMC8304960; doi:10.3390/cells10071834)
Supplement: Supplementary file 1 [file cells-10-01834-s001.zip › cells-1283814-supplementary.pdf]

cells MDPI

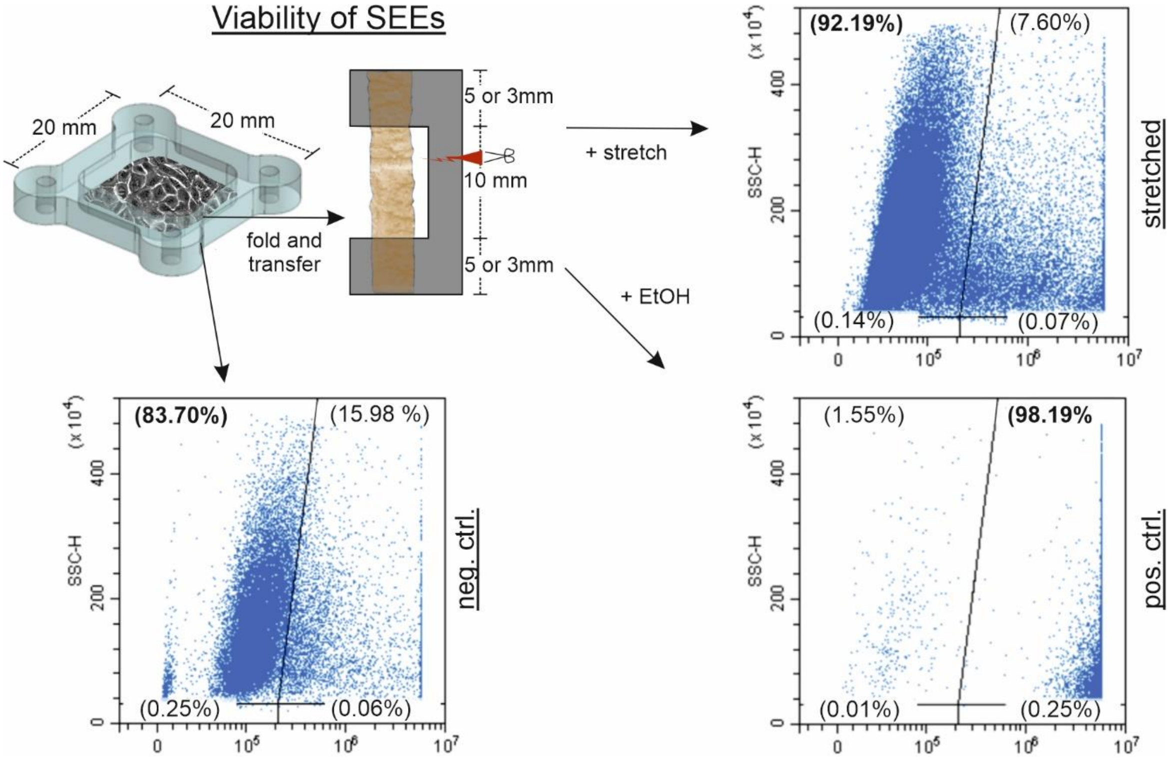

Figure S1. SEEs are still viable after long-term static stretch experiments: To prove biocompatibility of our experimental setup for SEEs, viability assays based on cell permeability and subsequent flow cytometry were performed. Cells denatured in EtOH served as positive control and non-transferred SEE as negative control.

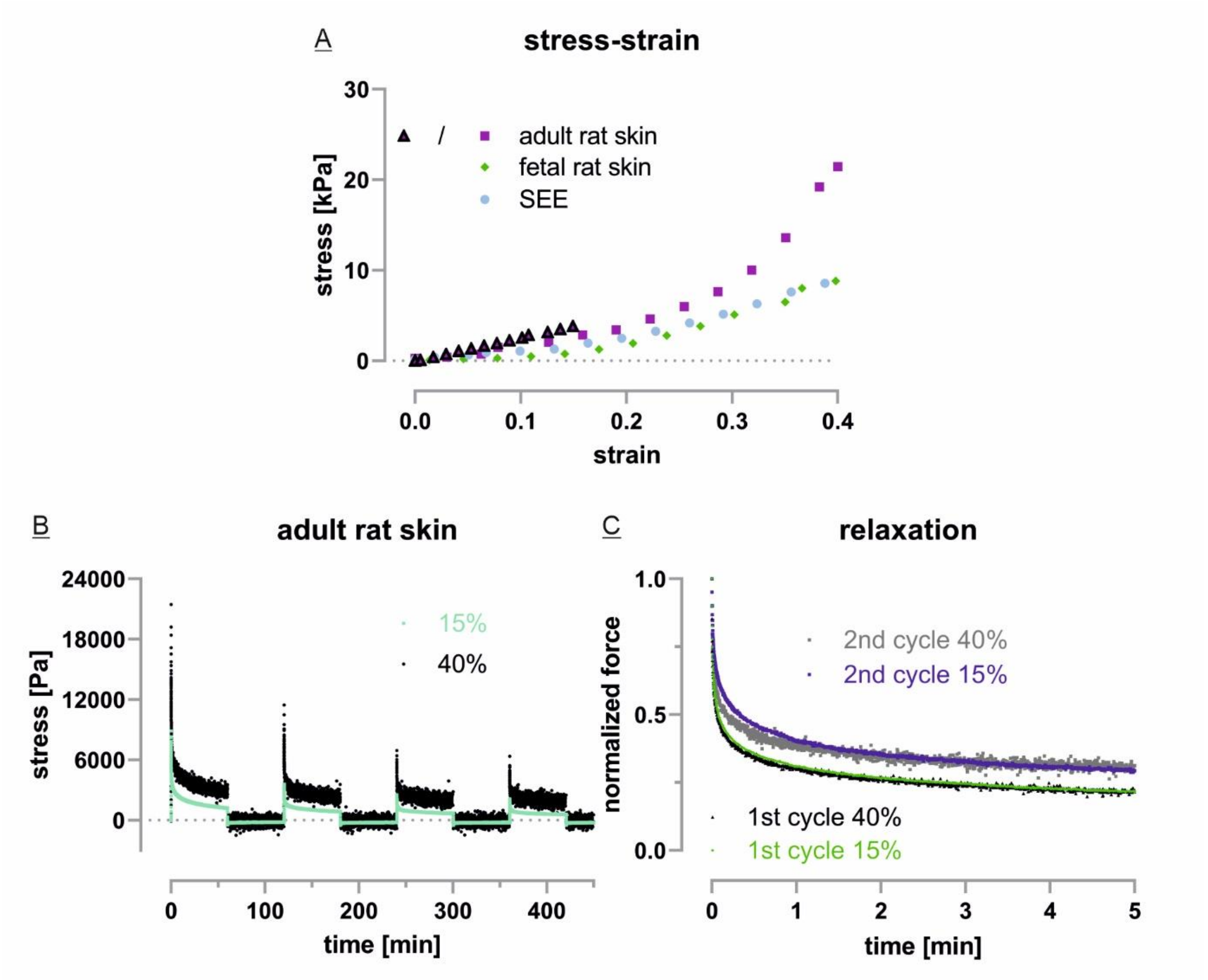

Figure S2. Adult skin responds similar to 15% and 40% of static stretch: **(A)** Tested tissue was either loaded with 3.2 mm/s to 40% (adult rat skin: purple squares, fetal rat skin: green diamonds and SEE: blue dots) or with 1.2 mm/s to 15% (adult rat skin: black bordered triangles) of its initial length and the tissue stress calculated from the estimated cross-sectional area (see Figure 3). Shown are representative stress-strain curves for every model system and stretch parameter. **(B)** To avoid experimental bias, adult rat skin was tested with an equal protocol of 40% amplitude as fetal rat skin and simplified epidermis equivalents (SEEs) of the same study. Shown are representative data of two independent experiments. Because of enhanced maximal force levels at 40% strain, a force transducer with a larger range (0-2 N) had to be used. A higher noise level was therefore inevitable. **(C)** Force values of the first and second stretch cycle were normalized to its initial peak force for both amplitudes and plotted within the first 5 min.

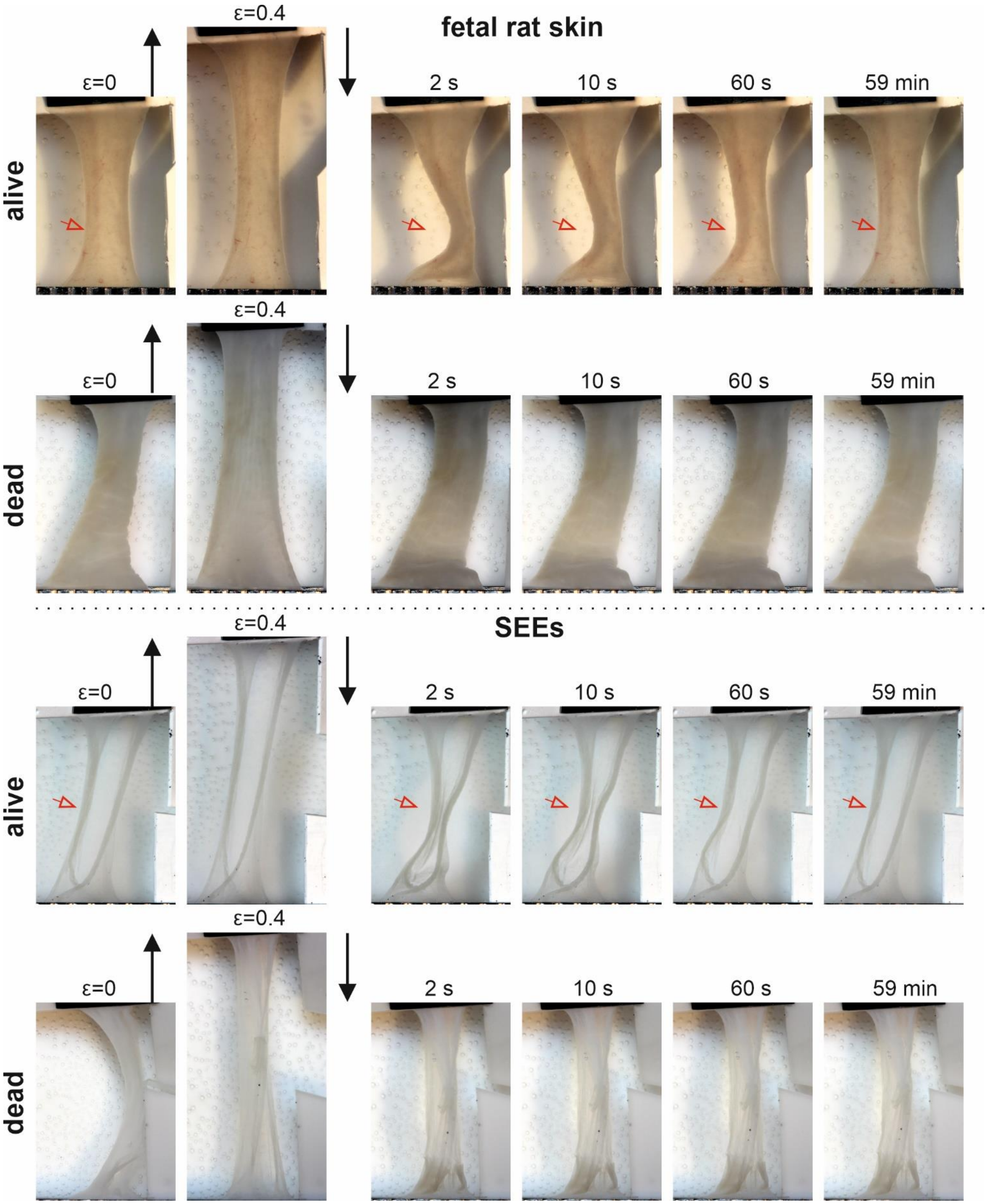

Figure S3. Tissue strain recovery is an active process: Images of the tissues were taken at indicated time points and strain levels. The fetal rat skin and SEEs were either directly stretched after isolation (alive) or incubated for 3 min in EtOH prior to stretch (dead). Epidermal viability was confirmed independently (**Figure 3**). Red arrowheads indicate locations of tissue bending. Note: Strong plastic deformation of dead fetal rat skin and SEEs occurred but is barely visible due to bending in z-direction.

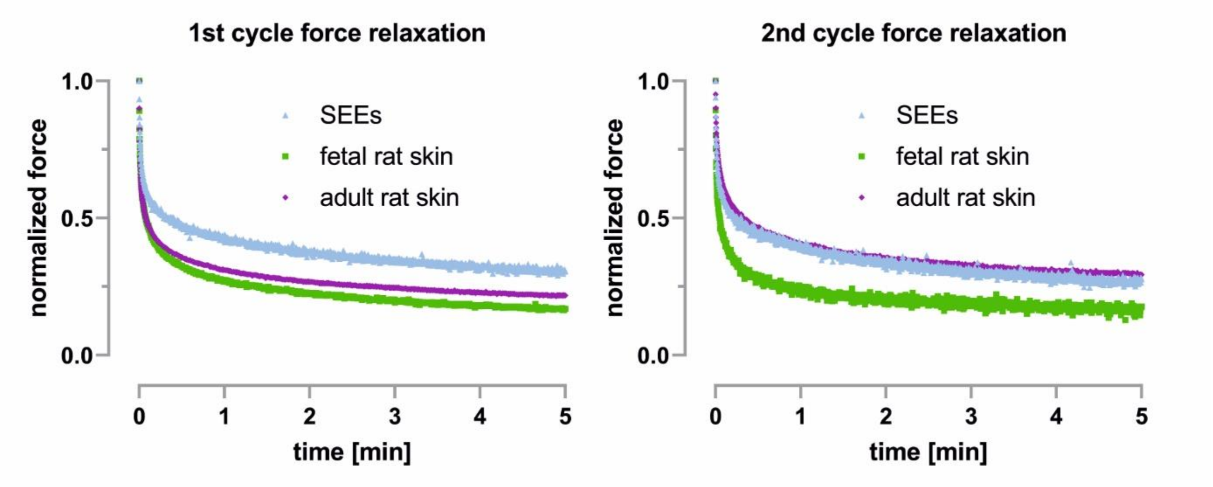

Figure S4. Multilayered SEEs mimic adult rat skin after preconditioning in first stretch cycle: Data replotted **from Figure 5 B-D** to allow comparison of tissue dependent force relaxation during first and second cycle of static stretch with 1 h rest in between the cycles. Data are normalized means (fetal: n=3, adult and SEE: n=4).
